# Adolescents, parents, and providers' experiences of triadic encounters in paediatric diabetes clinics: A qualitative study

**DOI:** 10.1111/hex.13916

**Published:** 2023-11-20

**Authors:** Imelda Coyne, Sinead Pembroke, Betsy Sleath, Maria Brenner, Edna F. Roche, Carol Hilliard, Declan Cody

**Affiliations:** ^1^ Trinity College Dublin The University of Dublin Dublin Ireland; ^2^ University of North Carolina at Chapel Hill Chapel Hill North Carolina USA; ^3^ University College Dublin Dublin Ireland; ^4^ Trinity College Dublin, Children's Health Ireland Tallaght University Hospital The University of Dublin Dublin Ireland; ^5^ Children's Health Ireland University College Dublin Dublin Ireland; ^6^ Children's Health Ireland at Crumlin Dublin Ireland

**Keywords:** adolescents, communication, interaction, out patient clinic, parents, providers, Type 1 diabetes

## Abstract

**Introduction:**

Adolescents with Type 1 diabetes are a cohort whose self‐management of their diabetes care often declines during adolescence which can lead to adverse health outcomes. Research indicates that providers find it challenging to engage adolescents in communication exchanges during triadic encounters in diabetes clinics. Our study aimed to explore adolescents, parents, and providers' experiences of clinic encounters.

**Methods:**

A qualitative study was conducted with a convenience sample of 13 adolescents with Type 1 diabetes (aged 11–17), 14 parents, and seven providers. Participants were recruited from two outpatient diabetes clinics in two urban children's hospitals, Ireland. Data were obtained using a combination of interviews and focus groups. Data were analysed thematically.

**Results:**

Adolescents and their parents appeared to hold both positive and negative experiences of diabetes clinic encounters. Providers reported challenges associated with engaging adolescents in communication exchanges. The structure, focus and style of clinic encounters created barriers that potentially led to suboptimal adolescent participation and impaired provider–adolescent communication during clinic visits.

**Conclusions:**

The findings provide insights into the challenges associated with adolescents' engagement in communication encounters in diabetes clinics. Healthcare providers could encourage adolescents to be more actively involved in their diabetes management, by taking an adolescent‐centred approach and creating a nonjudgemental milieu. Focusing on adolescent's agenda could lead to more meaningful and relevant discussions between providers and adolescents and ensure more tailored education in the time available. Adolescence is a risky period for nonadherence and adverse health complications; therefore, it is critical that providers make every contact count in diabetes clinic encounters.

**Patient or Public Involvement:**

The study's design and delivery were guided by two advisory groups, comprising (1) five adolescents living with Type 1 diabetes (T1D) and (2) five parents of an adolescent with T1D.

## 1 INTRODUCTION

Type 1 diabetes (T1D) is one of the most common chronic conditions among children worldwide with the incidence of adolescents with T1D increasing worldwide for several decades.^1^ Managing diabetes is extremely demanding as it is a complex metabolic disorder that involves frequent blood glucose monitoring, administering medication, balancing insulin requirements, following dietary guidelines and treating hypo or hyperglycaemia. Adolescence is a risky period because metabolic control tends to deteriorate partly due to pubertal insulin resistance and partly due to reduced adherence to diabetes regimen, assumption of risk taking behaviours and difficulties with taking on self‐management.^2^ Maintaining optimal diabetes care can be challenging because adolescents are striving for independence, negotiating identity and wanting to be the same as their peers.^3^ This can lead to poor glycaemic control, infrequent clinic attendance, loss to follow‐up, leading to increased risk of complications and adverse health outcomes.^2^ Furthermore, high proportions are lost to follow‐up or experiencing long gaps in care when they transition to adult services.^4^, ^5^, ^6^

Given the risks to adolescents' immediate and long‐term health of nonadherence, it is essential that adolescents actively participate in healthcare encounters^7^ so that they can develop skills in communication, self‐advocacy and build self‐management skills. Current international guidelines recommend that services are adolescent‐centred, and have a strong focus on enabling and empowering adolescents to ask questions and be actively involved in their diabetes care.^2^ Good relationships and effective communication between adolescents and providers promote engagement in diabetes encounters and encourages regular clinic attendance which helps with glycaemic control.^8^, ^9^

While good practices exist, research indicates that providers often fail to acknowledge and address adolescents' developmental and health‐related needs in clinic encounters.^7^ Furthermore adolescents often perceive clinic visits as stressful and negative, describing concerns about confrontation by providers regarding poor glycaemic control,^10^ and lack of two‐way conversation.^11^ In addition, communication is more complicated in paediatric clinics due to the interplay and competing communication needs of parent, adolescent and healthcare provider (HP). Studies of communication in triadic encounters have illustrated how providers mainly communicate with parents, have difficulty interacting adequately with adolescents^7^ resulting in adolescents taking a ‘backseat’^12^ and becoming ‘bystanders’.^13^, ^14^ Clearly more needs to be done to reorient the interaction towards the needs of adolescents who have responsibility for the lifelong management of their condition. Therefore, we explored adolescents, parents' and providers' experiences of participating in clinic encounters to identify current perceptions of clinic interactions. The results of this qualitative study were used to inform the development of an intervention to help adolescents to engage more in communication in clinic encounters.

## 2 METHODS

Using a qualitative design, focus groups (FGs) and individual interviews were held with adolescents aged 11–17 years, their parents and HPs, from diabetes clinics in two urban children's hospitals in Ireland. This study adhered to the consolidated criteria for reporting qualitative research guidelines. Ethical approval was received from both participating hospitals and the university research ethics committee. Information leaflets about the study were distributed by nurses in both clinics. Participants (adolescents, parents and HPs) in the clinics were recruited directly by the lead researcher during the conduct of the adolescent clinics. Informed written consent was obtained from all participants who agreed to participate. Participants could choose to attend an interview or a FG. During the recruitment phase, participants were offered a choice as to where they wanted to be interviewed, including in their homes or in a private room beside the hospital clinic. As prior work has shown adolescents' preferences for communication and attitudes towards clinic consultations were best researched outside the hospital setting.^13^

The interview guides focused on four key topics which were: (1) Experiences of attending the clinic, (2) What usually happens during the consultation, (3) Feelings and views about communication with providers and (4) Roles and behaviour during the encounters. Prompts were used to probe replies further when necessary. Interviews ranged from 30 to 70 min.

### 2.1 Data analysis

Interviews were audio‐recorded, transcribed verbatim and imported into the qualitative software programme NVivo V11.0.^15^ Using a method of constant comparison^16^ the data were coded line‐by‐line and grouped into themes^17^ by two researchers. The process of analysis consisted of (1) familiarisation with the transcripts, (2) generating initial codes, (3) collation of codes into key themes, (4) themes were reviewed by the researchers until consensus reached and (5) and then the final themes were written up. Data collection and analysis occurred concurrently. The coded data and the final list of themes were discussed among the team until consensus was reached that the themes accurately reflected the data.

## 3 RESULTS

A total of 10 participants (three adolescents, three parents and four providers) participated in three separate FGs while the remaining participants (*n* = 24) chose individual interviews. For adolescents who opted for a one‐to‐one interview, they found it easier because they felt self‐conscious when speaking in a group setting, especially if they had never spoken about their diabetes with their peers. For parents and HPs, the flexibility of individual interviews helped them to take part while managing their other commitments such as childcare and work. Most adolescents and parents chose to be interviewed in a private room in the university. This may have been due to concerns about anonymity and/or the central location of the university. Holding the interviews away from the hospital may have encouraged the adolescents and parents to speak more freely. The final sample consisted of 13 adolescents with T1D (aged 11–17), 14 parents and seven providers (see Table 1).

**Table 1:** Participant characteristics.

Parents ID	Gender	Adolescents ID	Age	Gender	Age when diagnosed with type 1 diabetes (years)
IP1	Female	IA1	16	Female	14
IP2	Female	IA2	14	Female	5
IP3	Female	IA3	16	Female	1
IP4	Female	IA4	13	Female	3
P5	Female	IA5	11	Female	1
P6	Female	IA6	14	Female	5
P7	Female	IA7	14	Female	13
P8	Female	IA8	11	Female	5
P9	Female	IA9	11	Male	10
P10	Female	IA10	13	Female	5
P11	Female	IA11	17	Male	6
P12	Female	IA12	12	Female	4
P13	Male	IA13	14	Male	3
P14	Female	Not applicable, caregiver interview only			

Providers ID	Gender	Profession	More than 10 years in clinical practice
HP1	Male	Doctor	Yes
HP2	Female	Doctor	Yes
HP 3	Female	Dietician	Yes
HP4	Female	Nurse	Yes
HP5	Female	Nurse	Yes
HP6	Female	Nurse	Yes
HP7	Female	Nurse	Yes

Abbreviation: HP, healthcare provider.

The results are presented under four themes: (1) Feelings about the structure of the clinic encounter, (2) Feelings about the focus of the clinic encounter, (3) Communication with providers; and (4) Adolescent and parent engagement during the clinic encounter. Codes used with statements denote: Parent (P), Adolescent (A), HPs, Interviews (I) and FGs.

### 3.1 Theme 1: Feelings about the structure of the clinic encounter

The structure of the clinic encounters usually involved seeing more than one provider starting with a nurse, usually followed by a junior doctor and then a consultant. If the consultant recommends it or the adolescent requests it, they may also see a dietician, psychologist and/or social worker:

So, you'd come in and they do your weight, height, HbA_1c_ (glycated haemoglobin), your blood sugars. And you'd see—not in any particular order, it depends—your diabetes nurse, the student doctor, the consultant and then sometimes the dietician or the psychologist as well. (A FG)

Consequently, the structure of the clinic visit was described by adolescents and parents as a slow process, as they could be at the clinic for 2–3 h. A lengthy waiting time was involved between seeing each provider, which contributed to increasing the overall time spent at the clinic, which often led to the final consultation with the consultant being rushed:

It's like a mix where they ask you questions but then they'd say is there anything you want to ask me, but they'd be quite rushed because it's so busy. They'd only have a few minutes to speak to you. (A FG)

Likewise, providers felt that the time was a barrier to engaging with adolescents and for adolescents feeling comfortable to engage:

The time factor, and everybody is mindful, I'm sure the young person is mindful that there's a clock on and that there's a queue of people outside. (IHP2)

The structure of the clinic visit was also repetitive. Parents and adolescents explained that each provider often asked the same questions, which was frustrating. Providers were aware that adolescents and parents could encounter different providers depending on the flow of the clinic and the needs of the adolescent. Parents and adolescents also mentioned that they usually saw a different doctor at each visit which made it challenging to form adolescent–provider relationships, and a consistent diabetes care plan:

You're seeing different people all the time. You're not seeing the same. That Is hard I think for them (adolescents) and for the person (doctor) because your kind of trying to pick up where someone else left off. (IP2)

### 3.2 Theme 2: Feelings about the focus of the clinic encounter

Discussions between adolescent, parent and doctor was described as largely focused on numbers and the medical side of diabetes:

It is more like medical stuff and like how your actual numbers are looking and how your blood results, and all that sort of thing. (IA3)

Anxiety and stress were reported by adolescents and parents, in relation to attending the diabetes clinic:

Really stressful because you feel kind of like, did I do everything right? So, that's why I would say it was stressful. I didn't like being there. (IA2)

Yeah, we dread it, we dread it, every time we go to clinic, we absolutely dread it. (IP2)

The stress and anxiety associated with the clinic visit appeared to be caused by the main focus of the clinic encounter being on the HbA_1c_ number:

We do HbA_1c_ at the start of the clinic because it's a really important thing to do, it's a really important discussion point, it gives people a sense of where things are going. It's not all about HbA_1c_ but it does give people a sense of what is happening. (IHP2)

Although providers said that the HbA_1c_ was not the main focus point, parents and adolescents reported feeling that it was. Thus, they reported feeling under pressure:

The HbA_1c_—everyone just fears what number will come out of the machine … the whole visit then depends on that. It feels a little bit like a test that you have to pass. (IP11)

Discussions around blood sugar levels was usually supported by online coloured graphs which one adolescent felt was helpful:

They like show you on the screen, like they've different colours, so … it lets you see where you need to kind of improve and like what times and what meals and stuff like, so it's really helpful. (IA1)

Whereas other adolescents and parents reported feeling nervous, stressed, and unhappy when confronted with the graphs. Some interpreted the discussion around the HbA1c as a blame game:

When I see all my reds and my bad colours, I'm like oops … you know you're gonna get in trouble over them. (IA11)

Well, I did think that as well, I thought that they were like criticising me. (IA4)

This leads to questioning from providers, with the aim of understanding patterns emerging from the graphs, in relation to the blood sugars. Some providers were aware that adolescents could be anxious and tried to explain the rationale for questions about levels and offer reassurance.

Probably a little nervous you know, especially, if their control isn't great, see it as a bad thing, so that probably affects them so we try and talk about that we're not here to blame or hate, we're here to support and try figure out answers and solutions if they are having issues, so trying to put it into that context. (IHP1)

However, some adolescents and parents felt this line of questioning was interrogative and repetitive, with little room for meaningful engagement which could be stressful.

Ask me the same questions, feel like I had to get them right … really stressful. (IA2)

One adolescent felt that some encouragement would help rather than blame when blood sugars are high.

I think that they should like praise you more. I think that the people in the clinic should … when you get HBA1C that's a bit higher you should just like be nicer to the person and stop like blaming them because I feel like when that happened to me, I felt really bad like when I was in the clinic. (IA2)

Most providers were aware that adolescents found attending the clinic difficult which created a barrier to engaging adolescents in communication interactions:

Often a clinical environment is very intimidating for young people. They've a million other places they want to be, so actually engaging them is challenging. (IHP2)

Providers also noted that adolescence was a difficult time as many adolescents just ‘want to park it’ (diabetes care) and forget about it (IHP1). Another provider noted that adolescents are a difficult group to engage, therefore it is essential to focus on the positives to encourage their engagement. Some parents spoke about having to reward or bribe their child to attend the diabetes clinic:

She hates going, the only way that I can get her to go is that I'll promise her something afterwards … it's kind of just a sweetener to make it just a bit easier for her. (IP2)

### 3.3 Theme 3: Communication with HPs

Despite some dissatisfaction with the structure and focus of the consultation, adolescents and parents generally felt that providers were helpful and professional. Providers reported that their style of communication was usually directed at the adolescent. This appeared to be a gradual approach, as both parents and adolescents noticed the conversation was directed more at the adolescent as they got older.

I'm normally kind of still kind of shy but like I, there's two consultants but I mainly speak to one of them and she like talks to me, she doesn't even look at my mam, she's talking like directly to me and then if I don't know anything I'll like look at my mam and she can tell. (IA4)

Adolescents spoke positively about this style of communication, because it allowed them to understand decisions around their diabetes care plan:

I like when they talk to me instead of mam because I understand I'm fourteen and I need to know what I'm doing. Often, I went home, and I didn't have a clue what they were talking about. (IA13)

However, for some adolescents this communication did not feel genuine, and they still felt that the conversation was directed at the parent which was also reported by some parents.

I have heard her (adolescent) describe the dynamic to other people and she would say they talk to me but then they really look at Mum, because I have all the answers. (IP4)

Adolescents reported feeling more comfortable speaking with nurses than with doctors. Reasons attributed to the ease and comfort communicating with nurses included the style and tone they use, remembering personal details and taking an interest in the adolescents' life outside of diabetes. Communication with nurses was described as relaxed, helpful and focused on building a relationship which made it easier for adolescents to ask questions and exchange information:

Because they are so friendly, I just get into a conversation with them, and we could talk for ages … like they are just really nice and if there is anything wrong, they tell me like if you want to ask me questions you can. (IA10)

One parent felt that her adolescent had a more positive relationship with nurses as the focus appeared to be on getting to know the adolescent rather than specifically information‐gathering.

He would have a much more positive relationship and he would engage with them (nurses) a bit more. I suppose he thinks they are more genuine really … They actually do have an interest in him they are not just writing it all down in a chart. And they would never sit there and take notes while they are talking to him; they would just talk to him. (IP11)

Furthermore, communication with nurses was not just conversational, it was also educational. Likewise, nurses spoke about the importance of focusing on the person rather than diabetes. In contrast, communication with doctors was described as more formal and focused on the medical aspects of diabetes care, which contributed to adolescents not feeling comfortable engaging:

Doctors would probably be less like normal conversations and more just down to the actual readings … basically they like tell you … where you're going wrong and stuff like that. (IA1)

The nurses are relaxed and casual. The doctor's a bit more formal. (IA13)

These feelings about the encounters with doctors could result in adolescents ‘switching off’ or opting out from the encounter as described by one parent:

He just literally lies back in the chair, doesn't make eye contact, doesn't engage with them at all … He just nods when he has to and that is about it. (IP11)

However, other adolescents and parents noted that some doctors do make casual conversation, and some communicated really well with adolescents. Likewise, doctors spoke about discussing adolescents' daily life to put them at ease and because of the impact of activities on diabetes control.

We do tend to talk a lot about school, we talk a lot about sport, and try and be reasonably cognisant of what's going on in the young person's life … because that has a massive impact in terms of diabetes related control, so it's not in a vacuum. (IHP2)

One adolescent suggested that doctors should make more conversations about informal nonmedical topics as this would make adolescents feel more comfortable to interact.

Kind of maybe like have it like more like personal, like even like questions like how is like your sports going, or things like that, just to make you more comfortable to introduce yourself and all that sort of thing, rather than it being like really like professional and like, even if it is, but it's just kind of easier if you feel more comfortable in the environment. (IA3)

### 3.4 Theme 4: Adolescents and parents' roles during the clinic encounter

Adolescents' engagement with providers varied. For most this appeared to be limited to answering questions, and not asking questions because of a lack of confidence and a fear of being wrong or feeling that they should know the answer already. Parents’ role and support were influencing factors in adolescent engagement with providers:

I think it depends if the parents are there; if the parents facilitate the communication or are a barrier to it. You always direct it towards the kids themselves, but obviously its age dependent and the input of the parents. But you will see the kids that will shy away and let the parents answer and the ones where you nearly have to quieten the parents and let the kids speak. (FG healthcare provider).

Parents' role in the clinic encounter appeared to vary according to the level of independence attributed to the adolescent in performing diabetes care‐related activities. Consequently, as the parent negotiates this role with the adolescent, this could impact on how they engage with the provider. At times, parents spoke about how they had become so used to caring for their adolescent's diabetes that they found it difficult not to interject or respond on their behalf:

You were nearly so used to it, you were nearly kind of saying, now don't say anything, and sit her in the first chair. Because you were just used to doing it for them. (IP1)

Support also meant ‘covering for’ the adolescent if s/he was caught out doing something that the provider may not be satisfied with:

Now I've tried to cover for her a little bit at clinic … because I have to try to support her. (IP2)

Parents reported feeling responsible for their adolescent's diabetes care, and some felt they needed to show that they were doing a good job and thus interjected or responded on the adolescent's behalf.

I suppose in one way you are hoping that they see that you are doing a good job because that is your like five minutes or whatever with you are kind of proving that you are doing your best. (IP11)

For some adolescents, having their parent present at the visit discouraged them from asking questions, particularly about risk‐taking issues, such as alcohol:

I wanted to ask more about the drink, but I didn't really ask because my mam was sitting there, and I was like should I ask, should I not but I didn't ask. (IA11)

Most adolescents appeared to prefer that their parents answered questions on their behalf. Reasons provided included being shy, not having enough time to give an answer and level of involvement in their diabetes care. They reported that they often relied upon their parents to supply relevant information. While providers felt that parents' contributions could be beneficial when adolescents were shy or quiet, they noted that parents could be overpowering and needed to take a ‘backseat’ in communication exchanges:

They're just not getting a chance for their voice to be heard because mam and dad are so used to doing everything, they've been in charge … sometimes parents can be overpowering. (FG healthcare provider)

Similarly, a parent noted that they while they felt it was important to encourage their adolescent to answer the doctors' questions when there was silence, they felt they had to interject with the sought information.

I probably am jumping in more than I would like to imagine. Because you just want to get all the information down and you know in a way you feel like you just want them to know that you are doing a good job. That you do feel like you are, not being judged but you want to show that you are doing a good job and you are keeping them healthy. (IP4)

Likewise, one adolescent suggested wanting time alone with their provider.

I'd rather like answer the questions or like just have the time … even like five minutes before or afterwards … like your parent could step outside the door or something like that. (IA3)

Other providers noted that parents' presence could be beneficial or a hindrance depending on the adolescent's personality and the situation:

Parents will butt in and stop them talking but at other time they may talk more freely without their parents but they also might just clam up and not talk at all, so it really depends on the adolescent, so some of them might come in themselves, and might tell their parents to stay out but sometimes you just don't know if you're getting the truth, sometimes its beneficial to have them there. (IHP3)

Some providers reported offering adolescents the choice to be seen alone while others felt that parents should stay involved and so they encouraged them to share the care with the adolescent so that the adolescent was supported during adolescence as it was a challenging time.

## 4 DISCUSSION

Adolescents and parents appeared to hold both positive and negative experiences of encounters in diabetes clinics. Our results showed that adolescents particularly valued communication and emotional support from nurses whilst encounters with doctors were perceived as more formal and rushed which has been reported in prior studies.^10^, ^11^ Nurses appeared to use an affective (socioemotional) style of communication whereas doctors appeared to use a more instrumental (task focused) style directed towards information‐gathering. When doctors used an affective style, it was often interpreted as not being genuine due to doctors' nonverbal behaviour (not listening or busy writing).

The structure, focus and style of clinic encounters clearly created barriers that potentially led to suboptimal adolescent participation and impaired provider–adolescent communication which has been reported before.^7^, ^18^, ^19^ Lengthy wait times coupled with the retelling of information to different providers was frustrating and potentially created a less than positive mind‐set among adolescents and parents. Limited time with the consultant endocrinologist appeared to inhibit adolescents from asking questions and raising concerns. Doctors tended to focus on blood sugar levels as they wanted to determine how best to help adolescents to stay within acceptable blood sugar ranges. But this focus appeared to make adolescents feel that doctors were more interested in the disease than in them as a person, even though they tried to engage them in affective talk. The time issue coupled with the doctor's focus on ‘good and bad’ blood sugar levels led to participants perceiving clinic encounters as stressful and negative which has been reported before.^10^, ^20^ Being confronted about poor glycaemic control led to some adolescents feeling criticised and parents feeling defensive of their parenting abilities.

Parents were often anxious about blood sugar levels as they felt it was an evaluation of their parenting skills as some likened it to ‘a test that had to be passed’. Hence some used the clinic encounter to convey their actions in maintaining the diabetes regime that is, doing a good job. These findings reflect the narrative of personal responsibility where if parents are unable to manage diabetes, they judge themselves as deficient at a personal level which has been reported elsewhere.^21^ Feeling judged/blamed could explain why some adolescents had to be persuaded/bribed to attend the clinic. It also led to nondisclosure of information by adolescents with their parents ‘covering for’ them by ensuring they were supportive of the adolescent's explanations to the provider.

Doctors noted that adolescents were a difficult group to communicate with because of their developmental stage and parents' presence. They acknowledged that adolescents could feel blamed with the focus on good and bad blood sugar levels, however, they were not fully cognisant of how the ‘critical professional gaze’ could strongly inhibit adolescents' participation and active engagement. For adolescents, trust and a sense of emotional safety is a prerequisite for open communication with providers^22^ and focusing on ‘bad numbers’ and identification of problems is arguably a deficit‐centred approach that does not contribute to creating a therapeutic milieu. Feeling blamed could lead to adolescents experiencing a sense of hopelessness which could lead to loss of motivation and possibly neglect of diabetes regime. Confronting or persuading have been identified as communication techniques that have quite a negative impact on adolescents' diabetes self‐care and HbA_1c_.^9^ Similarly feeling judged and reprimanded can lead to poor information recall, nondisclosure of information, disengagement and in extreme cases to nonattendance in diabetes clinics.^20^ This is problematic as providers need to keep adolescents actively involved in diabetes self‐management.

It seems that more could be done to improve the structure and style of the clinic encounters and communication exchanges so that contact with adolescents and parents is optimised and they receive quality care. Sharing data electronically and ensuring continuity of staff may reduce the need for repetition of information. While waiting, parents and adolescents could be advised to write down their questions/concerns in advance and the consultation could start with the focus on their concerns and from that base, providers could work collaboratively with them to identify how to maintain good diabetes control in a sensitive and nonjudgemental way. Caring for adolescents can be challenging given their developmental changes and fluctuating diabetes control,^19^ therefore, providers could benefit from adolescent‐centred communication skills training and enhanced awareness of how to frame health advice with sensitivity^9^, ^23^

Simple interventions, such as previsit videos^24^ and a question prompt list,^25^ which are easy to implement and disseminate, have been shown to help adolescents engage more during clinic encounters.^26^ Focusing on adolescents' agenda could lead to more meaningful and relevant discussions between providers and adolescents, make better use of the time available, ensure more tailored education and encourage adolescents to take an active role in their diabetes management.^7^ When adolescents participate actively in diabetes encounters, they are likely to receive better emotional and informational support which could contribute to treatment adherence.^18^ Research indicates that patient‐centred communication leads to patient empowerment and improved diabetes management for both adolescents and parents^27^ and good relationships with providers encourages adolescents active engagement in diabetes encounters, leads to regular clinic attendance and is linked to positive health outcomes.^8^

The parents in this study appeared to be actively involved in clinic encounters and their contributions were viewed by most providers as essential whilst others felt that parents hindered communication with adolescents. While research studies indicate that parental oversight is problematic when it is ‘over protective’,^5^ it is at the same time a strong predictor of adolescents' adherence to diabetes regimen.^28^, ^29^ These conflicting findings may be due to differing perceptions/needs, parenting style and adolescents temperament^30^ but there is general agreement that maintaining parental involvement in diabetes management during adolescence is critical.^29^, ^31^ Parents can find it challenging to balance responsibility whilst promoting their adolescent's autonomy and so providers could help by encouraging parents to gradually ‘step back’ and advising them on how to support their adolescent efforts to engage in clinic encounters. Providing split consultations could convey respect for adolescents' developing autonomy, facilitate open communication and help them to engage with providers and some research indicates that parents are supportive of adolescents having time alone with their provider.^32^

## 5 STRENGTHS AND LIMITATIONS

The findings from this small qualitative study were used to inform further workshops with adolescents with T1D and their parents to seek their views on how clinic encounters could be improved and to codesign an intervention to promote their engagement in clinic encounters.^24^, ^25^ Further research plans are to test out the intervention in a pilot randomised trial in two diabetes clinics.

With regard to limitations, the findings reflect participants' personal retrospective accounts and as such are open to recall bias. It is also a limitation that almost all participants in the study identified as female as communication preferences and experiences may vary by gender.^33^ The reason for the skewed female‐to‐male ratio was due to more refusals from adolescent boys than girls during the recruitment phase. Some of the reasons for refusal included being too shy, feeling anxious because they did not want to be at the clinic for their appointment, feeling frustrated with long wait time and not interested in talking about their experiences with a researcher. We aimed to include fathers, but most adolescents were accompanied by their mother, and the few fathers who did attend declined to participate or their son/daughter declined. The sample of HPs was quite small, and the research was only done in two urban clinics which further limits generalisability. It is also a limitation that we did not collect data on the diabetes characteristics of the sample, including glycaemic control and diabetes regimen. Interviews and FGs are two methods that may generate different types of data, however, there were no noticeable differences between the data gathered in interviews or FGs perhaps because participants had chosen the approach that they preferred. Further research combining video recording with interviews would help to reveal nonverbal communication in diabetes clinic encounters.

## 6 CONCLUSION

Despite the limitations, this study does provide important insights into how clinic encounters are experienced by adolescents and their parents. Clearly much more could be done to improve the structure and style of the clinic encounters and communication exchanges so that contact with adolescents and parents is optimised and they receive quality care. Adolescents and parents usually attend diabetes clinic appointments every 3–4 months, and these are opportunities where providers can foster adolescents' self‐efficacy and self‐management skills which are essential for lifelong management of T1D. Making every contact count is a key strategy in effective and successful chronic illness healthcare management in Ireland.^34^ With adolescence such a risky period for adolescents with T1D, characterised by non‐adherence and adverse health complications, it is critical that providers make every contact count, by creating a nonjudgemental milieu, eliciting concerns and building positive trusting relationships with adolescents and parents. Positive adolescent‐parent–provider relationships characterised by agenda setting, open communication, understanding and genuine concern may encourage adolescents to engage more, and feel more motivated to actively manage their diabetes care. All these actions are likely to result in more engaged adolescents and ultimately healthier adults living with diabetes.

## AUTHOR CONTRIBUTIONS

Imelda Coyne conceptualised and designed the study with the support of Betsy Sleath. Imelda Coyne, Betsy Sleath, Maria Brenner, Declan Cody, Edna Roche, Carol Hilliard planned the study. Imelda Coyne and Sinead Pembroke coordinated the study. Imelda Coyne, Sinead Pembroke and Maria Brenner collected data. Imelda Coyne and Sinead Pembroke analysed and interpreted data. Imelda Coyne and Sinead Pembroke drafted the initial manuscript. Imelda Coyne, Sinead Pembroke, Betsy Sleath, Maria Brenner, Declan Cody, Edna Roche and Carol Hilliard contributed to the writing, reviewing, editing and approval of the manuscript.

## CONFLICT OF INTEREST STATEMENT

The authors declare no conflict of interest.

## ETHICS STATEMENT

Ethical approval was received from both participating hospitals and the university Research Ethics Committee.

## Data Availability

The focus group and interview data (transcripts) that support the study conclusions are unavailable for public access because informed consent to share the complete transcripts outside of the research team was not obtained from the study participants.
